# Editorial: Peripheral Nerve Regeneration

**DOI:** 10.3389/fncel.2019.00464

**Published:** 2019-10-15

**Authors:** Giovanna Gambarotta, Stefania Raimondo, Esther Udina, James B. Phillips, Kirsten Haastert-Talini

**Affiliations:** ^1^Department of Clinical and Biological Sciences, University of Torino, Orbassano, Italy; ^2^Neuroscience Institute of the “Cavalieri Ottolenghi” Foundation (NICO), University of Torino, Orbassano, Italy; ^3^Department of Cell Biology, Physiology and Immunology, Centro de Investigación Biomédica en Red sobre Enfermedades Neurodegenerativas, Institute of Neurosciences, Universitat Autònoma de Barcelona, Bellaterra, Spain; ^4^Department of Pharmacology, UCL School of Pharmacy, University College London, London, United Kingdom; ^5^UCL Centre for Nerve Engineering, University College London, London, United Kingdom; ^6^Institute of Neuroanatomy and Cell Biology, Hannover Medical School, Hanover, Germany; ^7^Center for Systems Neuroscience (ZSN) Hannover, Hanover, Germany

**Keywords:** axotomy, nerve guide, nerve graft, nerve sheath tumor, Schwann cells, dorsal root ganglion neurons, motoneurons, functional recovery

The Research Topic entitled “Peripheral Nerve Regeneration” is focused on strategies to promote and improve the same. Injuries to peripheral nerves have variable causation such as metabolic or hereditary poly-neuropathies, nerve sheath tumors, or nerve trauma resulting from occupational or car accidents or combat activities. Although knowledge on the processes allowing peripheral nerve regeneration after degeneration or injury has been significantly increased over the last decades, and although (micro-)surgical methods have been refined considerably, more research is still needed to minimize consequences for patients and society. Treatment of acutely or chronically injured peripheral nerves is far from being optimal. Patients suffer from severe impairment of their quality of life due to continuous disabilities and their treatment is still affiliated with high socio-economic costs.

We are very grateful to the 123 authors from 14 different countries and five continents that contributed to the current Research Topic with seven review articles and 12 original research articles. These publications look from different angles into the current challenges for the field. For more than 100 years investigative surgeons and basic researchers have revealed solid knowledge on (1) key events orchestrating the process of peripheral nerve regeneration, and (2) basic mechanisms and pathways that need to be activated or blocked for axonal regeneration and correct reinnervation of peripheral target tissue. They have also discovered (3) cellular key players and how lineage reprogramming is generating them, and, not to forget, (4) surgical approaches and biomaterials supporting successful recovery.

This collection of articles adds insights to the aforementioned knowledge and also provides technical information worth considering in future research. In the following we first give an overview about the included review articles before shortly reporting on the original research articles.

Ronchi et al. analyzed the potential applications, advantages and limitations of the rat median nerve injury and repair model in pre-clinical research. They provide a synthetic overview of a variety of methods that can be applied in this model for assessing functional nerve regeneration and present informative tables about the research that has made use of this model. Also the article of Lovati et al. considers pre-clinical work performed in rat models. They performed a systematic review of the literature (from January 2007 to October 2017) regarding the different decellularization protocols used and aimed, through assessment of the results from histological and functional read-outs, to compare the effectiveness of the different nerve grafts. Boecker et al. reviewed the clinical relevance of chitosan derived nerve guides for short gap repair in human patients. They give an overview on the physiologic properties of chitosan in comparison to other nerve guide materials and discuss actual and future translation of chitosan into clinical practice.

In their article Duraikannu et al. review the regenerative capacity of adult neurons and novel intrinsic pathways that have impact on axonal regrowth, downstream from growth factor receptors. They thereby describe a novel approach to enhance the intrinsic growth of adult neurons, targeting neuronal inhibitors of regeneration, like tumor suppressor molecules, by means of non-viral siRNA. Orchestration of proteases and secretases during Wallerian degeneration and setting-up of a pro-regenerative environment is the focus of the article contributed by Pellegatta and Taveggia. They describe the role of different proteases and secretases in controlling, on a post-translational level, the activity of transmembrane proteins involved in peripheral nerve regeneration, among which the neurotrophin receptor p75^NTR^, the receptor Notch, the growth factor Neuregulin-1, and how this creates a permissive environment to allow successful regeneration of injured axons. Jessen and Mirsky review the remarkable plasticity of Schwann cells and the characteristics of the denervated repair Schwann cell population. Knowledge about their distinct properties and cell-type specific control mechanisms can potentially be used to foster the development of effective treatments, further increasing regenerative potential and maintaining repair capacity for the extended periods that are required for substantial nerve regeneration.

Neurotrophic factors and especially brain derived neurotrophic factor (BDNF) are known to modulate axonal regeneration. McGregor and English put a particular focus on discussing the role of BDNF in activity-dependent treatments to enhance regeneration such as electrical stimulation, exercise, and optogenetic stimulation. The authors further discuss the impact of a common single nucleotide polymorphism in the human *bdfn* gene, Val66Met, on the effectiveness of these treatments and emphasize the need for personalized regenerative medicine.

The original research articles included into this Research Topic, again, cover versatile aspects of peripheral nerve regeneration research.

The characteristic properties of the graft material will have an important impact on the outcome of functional recovery after nerve gap repair. This is not only true for bioartificial grafts, but also for autografts that are commonly harvested from sensory nerves and implanted into motor (mixed) nerves. In this context Hercher et al. evaluated regeneration-associated gene expression in homotopic or heterotopic 6 mm rat femoral nerve grafts. They describe evident spatiotemporal differences in the expression of some trophic factors and cell adhesion molecules between grafts from sensory and motor branches. Although differences in the capabilities of these grafts to sustain motor regeneration in their short gap repair model were minor, the paper contributes to the debate about the concerns of using sensory donor nerves as the gold standard to repair especially long nerve defects. The paper of Wilcox et al. considers a technical issue for going beyond rodent models in peripheral nerve research. For ethical and practical reasons, research of nerve injury and repair is focused to rodent models. Nevertheless, during reconstructive nerve repair it is sometimes possible to obtain human nerve samples. These samples are, however, exposed to the complex surgical environment, bringing them in contact with chemical and physical factors which are not present when sampling animal tissues in a research laboratory. With the aim of optimizing protocols for the efficient extraction of RNA for quantitative analysis, the authors characterized the effect of time delays before cryopreservation and the effect of contact with antiseptic surgical reagents on the quantity and quality of RNA isolated from human samples in comparison to rat nerve samples.

The efficacy of two novel bioartificial nerve grafts has been investigated by other groups in two different models, the 10 mm rat median nerve and the 10 mm rat sciatic nerve model. Dietzmeyer et al. report their analysis of a more bendable 2-chambered chitosan nerve guide. Bendability was increased by a corrugated outer wall and the chambers were formed by longitudinally introducing a perforated chitosan film to assist axonal outgrowth. The authors evaluated functional regeneration with three methods in the advanced rat median nerve model and demonstrate that the device represents a promising and innovative alternative for nerve repair in mobile body parts such as digits. Chato-Astrain et al. describe their attempt to produce a tissue engineered nerve graft from mesenchymal stem cells integrated into a nanostructured hydrogel. They evaluated their graft alone or introduced into clinically approved collagen type I nerve guides in the rat sciatic nerve model. The results presented suggest that the novel nanostructured fibrin-agarose bio-artificial nerve substitutes support nerve tissue regeneration and functional recovery to an extent similar to the autograft control.

As also discussed in the review by McGregor and English, already mentioned above, physical exercise has been shown to add positive effects on motor recovery after nerve transection and repair. In this context Arbat-Plana et al. investigated how physical exercise can modulate the synaptic stripping and other spinal changes that spinal motoneurons undergo after axotomy in a rat model of end-to-end sciatic nerve repair. They describe that in addition to the activity-dependent modulation of the BDNF system, noradrenergic projections of the *locus coerules* are important for some but not all the effects that exercise induces on the spinal cord after peripheral nerve injury. Besides physical exercise, also supplementation of specific nutrients is frequently discussed to support peripheral nerve regeneration. The paper of Li et al. investigated the effects of ascorbic acid (vitamin C), an essential micronutrient, on nerve regeneration *in vivo* in mice and *in vitro*. The study showed that ascorbic acid improved regeneration and functional recovery following sciatic nerve crush, promoted neurite outgrowth in dorsal root ganglion (DRG) neuron cultures, enhanced proliferation and migration behavior in primary Schwann cell cultures, and positively modulated the polarization of primary macrophages in culture toward the pro-regenerative phenotype.

Since sufficient peripheral nerve regeneration obviously depends on axonal outgrowth and its support by Schwann cells, we have included into this special issue also basic research papers reporting novel knowledge about these topics. The critical role of micro domains in cell membranes, specifically cholesterol-rich lipid rafts which cluster proteins and other molecules together, is explored by Roselló-Busquets et al. using a series of *in vitro* and mouse *in vivo* models. This study showed that cholesterol depletion disrupted lipid rafts, altered growth cone morphology, and resulted in increased axonal growth during development and after axotomy, with implications for both peripheral and central nervous system research.

It is widely accepted that the neurotrophin receptor p75^NTR^ is a central component in nerve regeneration, but since it is expressed in both neurons and glia and has been associated with a wide range of cellular behaviors its specific role remains elusive. Gonçalves et al. generated a conditional knockout mouse model to silence expression of p75^NTR^ specifically in Schwann cells, subsequently showing that Schwann cell p75^NTR^ is not critical for axonal regrowth or remyelination following crush injury, although its absence did reduce nerve conduction velocity.

Following injury, the axons and their terminals release mitochondrial DNA (mtDNA) fragments and proteins known as “mitochondrial damage-associated molecular patterns” (mtDAMPs). In their paper, Korimová et al. hypothesized that mtDAMPs might stimulate Schwann cells to put out cytoplasmic processes through the action of the corresponding receptors and tested their hypothesis *in vitro* in RT4-D6P2T schwannoma cells.

Schwann cell proliferation is an important component of the peripheral nerve injury response and Tan et al. explored the effect of inhibiting RhoA-subfamily GTPases with C3 transferase on this process, since RhoA inhibition is an established target used for improving neural regeneration. Using an *in vitro* model they showed that the C3 transferase CT04 suppressed primary Schwann cell proliferation, but that this was likely to be independent of the ROCK pathway and instead involved inactivation of the AKT pathway. Also related to the topic of Schwann cell proliferation, Gordon et al. analyzed the proliferative capacity of rat Schwann cells obtained from distal nerve stumps subjected to acute denervation of 7 days or chronic denervation of either 7 weeks or 17 months, showing *in vitro* that these cells retained their capacity to myelinate DRG neurites, although with a reduced capacity. They hypothesized that, although these Schwann cells retain their ability to respond to axonal signals and to elaborate myelin, their low numbers and their reduced capacity to proliferate might account, at least in part, for the poor functional recovery observed after delayed surgical repair of injured peripheral nerves.

The last, but not least, publication included into this Research Topic, is a contribution of Stratton et al. who elucidated the role of the microenvironment to sustain/suppress malignant peripheral nerve sheath tumors (MPNST) that usually lead to aggressive nerve resections. Therefore, strategies aimed to suppress proliferation of these tumors would limit the functional loss derived from these malignancies. The authors present a novel *in vitro* model for studying MPNST by using isolated adult rodent Schwann cells and their results strengthen the finding that balanced properties of the tissue environment are crucial for tumor-suppression and point out to the high importance of the endoneurial tissue in this context.

We, the guest editors, thank all authors one more time for their valuable contributions and hope that the readers of this Research Topic will, as much as we did, enjoy learning the expert opinions and excellent research results presented.

## Author Contributions

All the authors have contributed to this editorial, writing comments to the different articles. KH-T has connected them all within the Editorial.

### Conflict of Interest

The authors declare that the research was conducted in the absence of any commercial or financial relationships that could be construed as a potential conflict of interest.

